# An Improved Two-Dimensional Direction-Of-Arrival Estimation Algorithm for L-Shaped Nested Arrays with Small Sample Sizes

**DOI:** 10.3390/s19092176

**Published:** 2019-05-10

**Authors:** Xiaofeng Gao, Xinhong Hao, Ping Li, Guolin Li

**Affiliations:** Science and Technology on Electromechanical Dynamic Control Laboratory, School of Mechatronical Engineering, Beijing Institute of Technology, 5 South Zhongguancun Street, Haidian District, Beijing 100081, China; nmbtgxf@bit.edu.cn (X.G.); liping85@bit.edu.cn (P.L.); 7920161015@bit.edu.cn (G.L.)

**Keywords:** 2-D DOA estimation, L-shaped nested arrays, small numbers of samples, cross-correlation matrix

## Abstract

In this paper, an improved two-dimensional (2-D) direction of arrival (DOA) estimation algorithm for L-shaped nested arrays is proposed. Unlike the approach for a classical nested array, which use the auto-correlation matrix (ACM) to increase the degrees of freedom (DOF), we utilize the cross-correlation matrix (CCM) of different sub-arrays to generate two long consecutive virtual arrays. These acquire a large number of DOF without redundant elements and eliminate noise effects. Furthermore, we reconstruct the CCM based on the singular value decomposition (SVD) operation in order to reduce the perturbation of noise for small numbers of samples. To cope with the matrix rank deficiency of the virtual arrays, we construct the full rank equivalent covariance matrices by using the output and its conjugate vector of virtual arrays. The unitary estimation of signal parameters via rotational invariance technique (ESPRIT) is then performed on the covariance matrices to obtain the DOA of incident signals with low computational complexity. Finally, angle pairing is achieved by deriving the equivalent signal vector of the virtual arrays using the estimated angles. Numerical simulation results show that the proposed algorithm not only provides more accurate 2-D DOA estimation performance with low complexity, but also achieves angle estimation for small numbers of samples compared to existing similar methods.

## 1. Introduction

As an important field of array signal processing, direction of arrival (DOA) estimation has been applied in a wide range of applications such as wireless communications, sonar, radar, and acoustic localization [1,2,3]. Furthermore, signal sources location and identification are essential for monitoring the electromagnetic environment, and several different technologies based on parameter estimation have been applied to it [4,5,6,7]. The key problem of parameter estimation is estimating the azimuth and elevation angles. To obtain these angles, several DOA estimation methods have been proposed, such as multiple signal classification (MUSIC) [8], Time-Reversal MUSIC (TR-MUSIC) [9,10,11], Root-MUSIC [12], estimation of signal parameters via rotational invariance technique (ESPRIT) [13], and so on. Compared with 1-D DOA estimation, the study of 2-D DOA estimation, which can obtain the azimuth and elevation angles simultaneously, has attracted much attention. Numerous high-resolution 2-D DOA estimation methods with different structured arrays have been proposed, including the two-parallel arrays [14,15], circular arrays [16,17], uniform rectangular arrays [18,19,20], and L-shaped arrays [21,22,23,24,25,26,27,28,29,30]. Due to the wider coverage area and lower Cram’er-Rao boundary (CRB) [31], great efforts have been focused on 2-D DOA estimation for L-shaped arrays.

Due to the geometric configuration of L-shaped arrays, the elevation and azimuth angles can be estimated independently from each linear arrays. As the classical subspace method, MUSIC can be directly employed to the received data matrix of L-shaped arrays [21], but 2-D pseudo-spectrum searching of MUSIC requires a large number of calculations. An algorithm based on the propagator method (PM) has been proposed to estimate the angles with two L-shaped arrays [22], but the algorithm needs an extra pair-matching method. Consequently, a pair-matching algorithm has been proposed by utilizing the diagonal factors of the cross-correlation matrix (CCM) [23]. To avoid pair-matching of azimuth and elevation angles, an estimation algorithm based on joint singular value decomposition (JSVD) has been proposed to obtain the auto-matched azimuth and elevation angles [24]. However, the beamforming-like operation of the algorithm requires heavy calculations. By employing the shift-invariance property of the CCM, an algorithm based on ESPRIT has been proposed [25], which performs eigenvalue decomposition (EVD) on improved CCMs to estimate the angles. Considering the properties of several CCMs with the same signal subspace, a subspace-based algorithm without singular value decomposition (SVD) or EVD has been proposed to obtain the azimuth and elevation angles [26]. Although this algorithm can acquire the signal subspace without SVD or EVD, the algorithm needs to minimize a cost function, which requires heavy calculations. In order to expand the array aperture and snapshots, the researchers have proposed an estimation algorithm based on the conjugate symmetry property of the uniform array’s manifold matrix [27]. However, the expanded snapshots only benefit the azimuth or elevation angles. Nie, X. [28] has presented a 2-D DOA estimation algorithm for closely spaced sources, which performs SVD on two matrices constructed by CCMs to estimate the azimuth and elevation angles. Pair-matching is achieved by using the conjugate property of the non-zero eigenvalues of two matrices. To reduce the complexity of 2-D DOA estimation, a novel auto-matched PM for L-shaped array has been presented [29]. Unfortunately, the signal subspace is obtained directly from CCM, and thus the performance of the method is affected by the residual correlations of noise vectors. Liang, J. [30] has proposed a joint 2-D DOA estimation algorithm, which constructs a new angle with the elevation and azimuth angle and divides the arrays into two sub-arrays. The ESPRIT algorithm is then performed to obtain the azimuth and elevation angles without additional pair-matching.

All of the algorithms mentioned above are applied to the L-shaped uniform arrays, which confine the aperture and the maximum number of incident signal sources to be estimated. In contrast to the uniform arrays, sparse arrays with more degrees of freedom (DOF) and larger apertures have aroused the interest of scholars and researchers, such as minimum redundancy arrays [32], co-prime arrays [33], and nested arrays [34]. Co-prime arrays have been proposed for increasing the DOFs and improving the estimation resolution. However, the co-arrays of co-prime arrays are usually not a filled uniform, which may lead to phase ambiguity. To make the utmost of the co-arrays, an off-grid method has been proposed [35], which interpolates the additional sensors to the generated co-arrays to convert the non-uniform arrays into uniform linear arrays (ULA). Zhou, C.W. [36] has proposed an estimation method based on virtual arrays interpolation to reconstruct the covariance matrix of co-arrays, which can utilize all the derived co-arrays. Compared with other sparse arrays, nested arrays with *N* physical sensors can generate $O(N^{2})$ DOF with a closed-form expression. Therefore, several algorithms based on sparse L-shaped arrays have been proposed for 2-D DOA estimation. Jian-F. G. [37] has proposed a joint 2-D DOA estimation algorithm for the L-shaped sparse arrays, that are composed of one uniform linear arrays and one sparse linear arrays (SLA). In order to obtain the auto-matching angles, the elevation angle is estimated first, and the azimuth angle is estimated using the estimated elevation angle. The resolution of the elevation angle estimated by the ULA is not as high as the azimuth angle estimated by the SLA. To make use of the advantages of nested arrays, an interlaced double precision 2-D DOA estimation algorithm using L-shaped nested arrays has been presented [38]. However, the azimuth or elevation angle is achieved based on solving a least-squares problem with another estimated angle. Influenced by noise perturbations, high-precision estimation can only be obtained for either the azimuth or elevation angle. By utilizing the spatial-temporal property of signals, Dong, Y.Y. [39] has proposed an algorithm for L-shaped nested arrays, which constructs several CCMs with different time lags and performs a signal subspace joint diagonalization technique (SSJD) to estimate the azimuth and elevation angles simultaneously. However, the number of valid snapshots is reduced by dividing output dates into several frames, and the SSJD technique requires a loop iterative operation which requires heavy calculations.

In this paper, we propose an improved algorithm for L-shaped nested arrays in order to estimate the azimuth and elevation angles with a small number of snapshots. The array configuration is composed of two orthogonal nested arrays in the X-Y plane. Rather than using the auto-correlation matrix (ACM) of nested arrays, we calculate the CCM using the output of different sub-arrays, which removes the noise vector. Considering the perturbation of the sample cross-correlation matrix under a small number of snapshots, we reconstruct the CCM by using SVD. The sub-matrices of the reconstructed CCM are used to generate two large consecutive virtual arrays without an overlap element or residual noise. Since the equivalent received signals of virtual arrays behave like coherent sources, the output of the virtual arrays and their conjugate vectors are used to construct the full rank Toeplitz matrix as the equivalent covariance matrix. This process overcomes the rank deficiency of the virtual arrays. To reduce the computational complexity of the algorithm, a unitary ESPRIT method is performed on the equivalent covariance matrix to estimate the azimuth and elevation angles, separately. Since the azimuth and elevation angles are obtained, we achieve the pair-matched angles by deriving the equivalent source vector of the virtual arrays with the estimated angles. Our procedure can also estimate the power of the incident signals. Detailed analysis and numerical simulation are provided to demonstrate the lower complexity and better performance of the proposed algorithm.

The rest of the paper is organized as follows. The array configuration and signal model are illustrated in Section 2. A description of the proposed algorithm is introduced in Section 3. The simulation results and analysis are shown in Section 4, and the conclusion is presented in Section 5.

The following notations will be used throughout the paper. Vectors and matrices are denoted by using lower-case and capital bold letters, respectively. The superscripts ${\left(\cdot \right)}^{*}$, ${\left(\cdot \right)}^{T}$, ${\left(\cdot \right)}^{+}$, and ${\left(\cdot \right)}^{H}$ represent the conjugate, transpose, pseudo-inverse, and conjugate transpose operations, respectively. The symbol ⊙ denotes the Khatri-Rao product between two matrices, and vec(⋅) denotes the column vectorization operator, which turns a matrix into a column vector. The symbol E(⋅) represents the expectation operator. Finally, the notation $\prod_{N}$ denotes an N×N exchange matrix with ones on its anti-diagonal and zeros elsewhere.

## 2. Array Configuration and Signal Model

**Figure 1:**
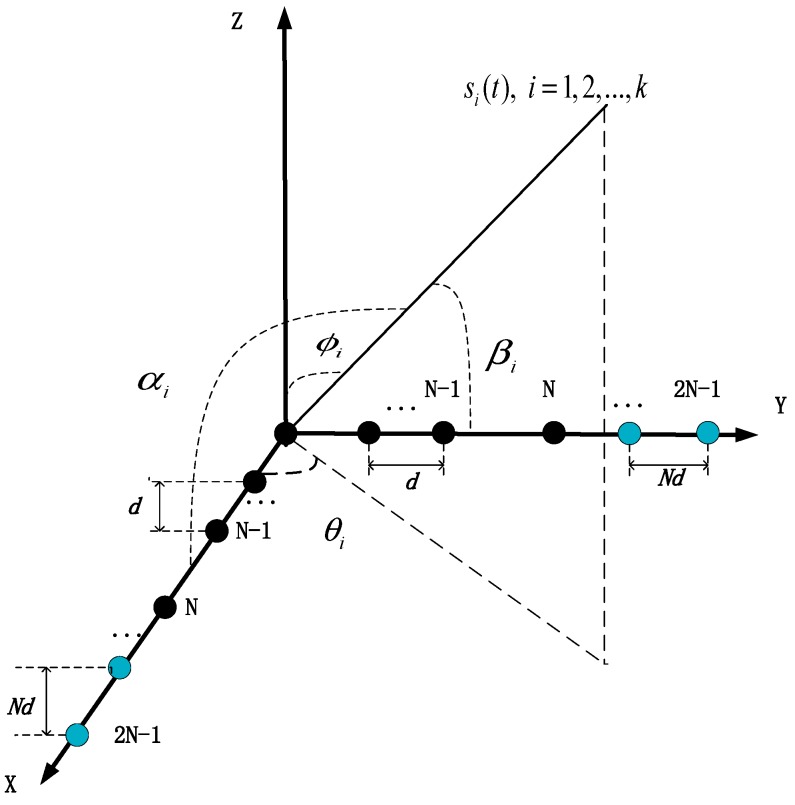
The geometry of the L-shaped nested array. Black dots indicate sensors of inner ULA with inter-spacing *d*, while blue dots show sensors of outer ULA with inter-spacing *Nd*.

The array geometric configuration is illustrated in Figure 1; the L-shaped nested arrays are composed of two orthogonal nested arrays in the X-Y plane. Each nested arrays consists of two ULAs, where the inner ULA contains N sensors with inter-sensor spacing d and the outer ULA contains N−1 sensors with inter-sensor spacing Nd, where d=1/2λ, and λ is the wavelength of the incident signal. The sensor at the origin is seen as the reference sensor. All sensors are assumed to be identical, omnidirectional, and isotropic. Suppose that there are K far-field, uncorrelated, narrowband signals $s_{i}(t)\;(i=1,\ldots ,K)$, impinging from distinct directions. Note that there are two ways to define the incident angles of sources. The first way is in terms of the azimuth angle $\theta_{i}$, measured between the projection of *i*-th incident signal in the X-Y plane and the X-axis, and the elevation angle $\phi_{i}$, measured between the *i*-th incident signal and the Z-axis. The second set of angles included the angle $\alpha_{i}$, which is measured between the *i*-th incident signal and the X-axis, and the angle $\beta_{i}$, which is measured between the *i*-th incident signal and the Y-axis. It is easy to verify that a relationship between the angles $\left(\alpha_{i},\beta_{i}\right)$ and the angles $\left(\theta_{i},\phi_{i}\right)$ exists, as shown in:

(1) $$\begin{array}{l} \cos\alpha_{i}=\cos\theta_{i}\sin\phi_{i}\\ \cos\beta_{i}=\sin\theta_{i}\sin\phi_{i} \end{array}$$

The array output vectors at the sub-array along the X-axis and Y-axis are written in matrix form as follows:

(2) $$\begin{array}{ll} X(t) & ={\left[x_{1}(t),x_{2}(t),\ldots ,x_{2N-1}(t)\right]}^{T}\\ & =A_{x}(\alpha )S(t)+N_{x}(t) \end{array}$$

(3) $$\begin{array}{ll} Y(t) & ={\left[y_{1}(t),y_{2}(t),\ldots ,y_{2N-1}(t)\right]}^{T}\\ & =A_{y}(\beta )S(t)+N_{y}(t) \end{array}$$

where $A_{x}(\alpha )=[a_{x}(\alpha_{1}),a_{x}(\alpha_{2}),\ldots ,a_{x}(\alpha_{k})]$, $A_{y}(\beta )=[a_{y}(\beta_{1}),a_{y}(\beta_{2}),\ldots ,a_{y}(\beta_{k})]$ are the (2N−1)×K array manifold matrices of the X-axis and Y-axis, respectively, and the *i*-th manifold vectors $a_{x}(\alpha_{i})$ and $a_{y}(\beta_{i})$ are expressed as follows:

(4) $$\begin{array}{ll} a_{x}(\alpha_{i})= & [1,e^{-j\pi \cos\alpha_{i}},\ldots ,e^{-j\pi (N-1)\cos\alpha_{i}},\\ & e^{-j\pi (2N-1)\cos\alpha_{i}},\ldots ,e^{-j\pi (N^{2}-1)\cos\alpha_{i}}{]}^{T} \end{array}$$

(5) $$\begin{array}{ll} a_{y}(\beta_{i})= & [1,e^{-j\pi \cos\beta_{i}},\ldots ,e^{-j\pi (N-1)\cos\beta_{i}},\\ & e^{-j\pi (2N-1)\cos\beta_{i}},\ldots ,e^{-j\pi (N^{2}-1)\cos\beta_{i}}{]}^{T} \end{array}$$

Additionally, $S(t)={\left[s_{1}(t),s_{2}(t),\ldots ,s_{k}(t)\right]}^{T}$ is the source signal vector, $N_{x}(t)={\left[n_{x1}(t),n_{x2}(t),\ldots ,n_{x2N-1}(t)\right]}^{T}$ and $N_{y}(t)={\left[n_{y1}(t),n_{y2}(t),\ldots ,n_{y2N-1}(t)\right]}^{T}$ are the additive noise vectors. In this paper, we suppose the incident signals with variances $\left[\sigma_{1}^{2},\sigma_{2}^{2},\ldots ,\sigma_{k}^{2}\right]$ are uncorrelated to each other, the additive noise is temporally and spatially complex white Gaussian noise with zero mean and variance $\sigma_{n}{}^{2}$, which are statistically independent of the signals. The number of incident signals k has already been estimated by a number detection technique. These properties of the source signal vector and additive noise vectors can be written as:

(6) $$E[S(t)S^{H}(t)]=diag(\sigma_{1}^{2},\sigma_{2}^{2},\ldots ,\sigma_{k}^{2})$$

(7) $$E[S(t)N_{i}{}^{H}(t)]=0$$

(8) $$E[N_{i}(t)N_{j}^{H}(t)]=\left\{ \begin{array}{ll} \sigma_{n}{}^{2}I & i=j\\ \;0 & i\neq j \end{array}\right.,$$

where I and 0 indicate identity and null matrices, respectively.

## 3. The Proposed Method

### 3.1. Constructing the Cross-Correlation Matrix for a Small Number of Snapshots

**Figure 2:**
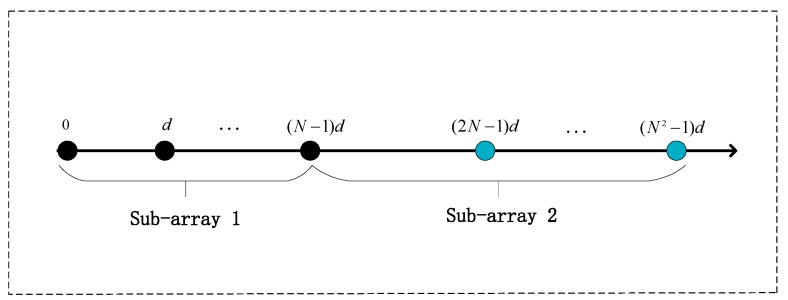
Subarray selection from the nested arrays of the X-axis. Black dots indicate sensors of sub-array 1 with inter-spacing *d*, while blue dots show sensors of sub-array 2 with inter-spacing *Nd*, the *N*-th sensor is shared by both sub-arrays, symbol denotes the location of sensor.

We divide the nested arrays of the X-axis into two subarrays, as shown in Figure 2. Since the arrays along the X-axis and Y-axis have similar structures, the sub-array division of Y-axis is analogously divided. The nested array along the X-axis is composed of 2*N*−1 sensors, there are *N* sensors in each sub-array, where the *N*-th sensor is shared by both sub-arrays. Sub-array 1 is a uniform linear array containing *N* sensors with spacing $d_{1}=\lambda /2$, and sub-array 2 is a uniform linear arrays contained *N* sensors with spacing $d_{2}=N\lambda /2$. The output vectors of sub-array 1 and sub-array 2 are given respectively by:

(9) $$\begin{array}{l} X_{1}(t)={\left[x_{1}(t),x_{2}(t),\ldots ,x_{N}(t)\right]}^{T}\\ =A_{x1}(\alpha )S(t)+N_{x1}(t) \end{array}$$

(10) $$\begin{array}{l} X_{2}(t)={\left[x_{N}(t),x_{N+1}(t),\ldots ,x_{2N-1}(t)\right]}^{T}\\ =A_{x2}(\alpha )S(t)+N_{x2}(t) \end{array}$$

where $N_{x1}(t)$ and $N_{x2}(t)$ are the noise vectors of sub-array 1 and sub-array 2, respectively. In addition, $A_{x1}(\alpha )=[a_{x1}(\alpha_{1}),a_{x1}(\alpha_{2}),\ldots ,a_{x1}(\alpha_{k})]$ and $A_{x2}(\alpha )=[a_{x2}(\alpha_{1}),a_{x2}(\alpha_{2}),\ldots ,a_{x2}(\alpha_{k})]$ denote the manifold matrices of sub-array 1 and sub-array 2, respectively, where the *i*-th column of these two manifold matrices are written as

(11) $$a_{x1}(\alpha_{i})={\left[1,e^{-j\pi \cos\alpha_{i}}\ldots ,e^{-j(N-1)\pi \cos\alpha_{i}}\right]}^{T}$$

(12) $$a_{x2}(\alpha_{i})={\left[e^{-j(N-1)\pi \cos\alpha_{i}},\ldots ,e^{-j(N^{2}-1)\pi \cos\alpha_{i}}\right]}^{T}$$

Similarly, sub-array 3 along the Y-axis is a uniform linear array containing *N* sensors with spacing $d_{1}=\lambda /2$, and the sub-array 4 along the Y-axis is a uniform linear array containing *N* sensors with spacing $d_{2}=N\lambda /2$, the output vector can be written as:

(13) $$\begin{array}{ll} Y_{1}(t) & ={\left[y_{1}(t),y_{2}(t),\ldots ,y_{N}(t)\right]}^{T}\\ & =A_{y1}(\beta )S(t)+N_{y1}(t) \end{array}$$

(14) $$\begin{array}{ll} Y_{2}(t) & ={\left[y_{N}(t),y_{N+1}(t),\ldots ,y_{2N-1}(t)\right]}^{T}\\ & =A_{y2}(\beta )S(t)+N_{y2}(t) \end{array}$$

where $N_{y1}(t)$ and $N_{y2}(t)$ are the noise vectors of sub-arrays 3 and 4, respectively, $A_{y1}(\beta )=[a_{y1}(\beta_{1}),a_{y1}(\beta_{2}),\ldots ,a_{y1}(\beta_{k})]$ and $A_{y2}(\beta )=[a_{y2}(\beta_{1}),a_{y2}(\beta_{2}),\ldots ,a_{y2}(\beta_{k})]$ are the manifold matrices of sub-array 3 and 4, respectively, where the *i*-th column of these two manifold matrices are written as:

(15) $$a_{y1}(\beta_{i})={\left[1,e^{-j\pi \cos\beta_{i}}\ldots ,e^{-j\pi (N-1)\cos\beta_{i}}\right]}^{T}$$

(16) $$a_{y2}(\beta_{i})={\left[e^{-j(N-1)\pi \cos\beta_{i}},\ldots ,e^{-j(N^{2}-1)\pi \cos\beta_{i}}\right]}^{T}.$$

By constructing the vectors with the outputs of the subarrays $Z_{1}(t)={\left[X_{1}(t),Y_{1}(t)\right]}^{T}$ and $Z_{2}(t)={\left[X_{2}(t),Y_{2}(t)\right]}^{T}$, the CCM of the two vectors can be computed as:

(17) $$R_{z}=E[Z_{1}(t)Z_{2}^{H}(t)]=\left[\begin{array}{l} X_{1}(t)\\ Y_{1}(t) \end{array}\right]{\left[\begin{array}{l} X_{2}(t)\\ Y_{2}(t) \end{array}\right]}^{H}=\left[\begin{array}{l} R_{x1x2}\;\;R_{x1y2}\\ R_{y1x2}\;\;R_{y1y2}\; \end{array}\right].$$

According to the Equation (17), the submatrices of $R_{z}$ can be expressed as:

(18) $$R_{x1x2}=E[X_{1}(t)X_{2}^{H}(t)]=A_{x1}(\alpha )R_{s}A_{x2}^{H}(\alpha )+\sigma_{n}{}^{2}R_{n0}\approx A_{x1}(\alpha )R_{s}A_{x2}^{H}(\alpha )$$

(19) $$R_{x1y2}=E[X_{1}(t)Y_{2}^{H}(t)]=A_{x1}(\alpha )R_{s}A_{y2}^{H}(\beta )$$

(20) $$R_{y1x2}=E[Y_{1}(t)X_{2}^{H}(t)]=A_{y1}(\beta )R_{s}A_{x2}^{H}(\alpha )$$

(21) $$R_{y1y2}=E[Y_{1}(t)Y_{2}^{H}(t)]=A_{y1}(\beta )R_{s}A_{y2}^{H}(\beta )+\sigma_{n}{}^{2}R_{n0}\approx A_{y1}(\beta )R_{s}A_{y2}^{H}(\beta ),$$

where $R_{s}=E[S(t)S^{H}(t)]=diag[\sigma_{1}^{2},\sigma_{2}^{2},\ldots ,\sigma_{k}^{2}]$, and $\sigma_{k}^{2}$ denotes the power of the *k*-th signal source. Since the noise vectors of different sensors are spatially independent and uncorrelated, the elements of $R_{n0}$ are all zeros except for the element $R_{n0}(N,1)=1$. Considering that the matrix $\sigma_{n}{}^{2}R_{n0}$ is an extremely sparse matrix, with only one non-zero element, it has little effect on the cross variance matrix $R_{z}$, so we omit it here and in the coming analysis. The noise term $\sigma_{n}{}^{2}R_{n0}$ is omitted only for simplifying analysis. The effect of omitting noise term $\sigma_{n}{}^{2}R_{n0}$ on DOA estimation is examined in Section 4. From Equations (18)–(21), the noise vector is removed by the cross-correlation operation.

We perform SVD on $R_{z}$, which can be expressed as:

(22) $$R_{z}=[U_{S}U_{N}]\left[\begin{array}{ll} \Lambda_{S} & \;0\\ \;0\; & \Lambda_{N} \end{array}\right]{\left[V_{S}\;V_{N}\right]}^{H}.$$

According to the second-order statistical properties of array signals, $\Lambda_{S}$ and $\Lambda_{N}$ are rectangular diagonal matrices with the *K* large singular values and other small singular values on the diagonal; $U_{S}$, $U_{N}$, $V_{S}$, and $V_{N}$ are unitary matrices whose columns are left-singular vectors and right-singular vectors corresponding to $\Lambda_{S}$ and $\Lambda_{N}$. Considering the noise vectors are removed by the cross-correlation operation, $\Lambda_{N}$ should be the null matrix.

In practice, the CCM $R_{z}$ is replaced by the sample CCM ${\hat{R}}_{z}$. Assuming that *L* snapshots are available, the sample CCM matrix ${\hat{R}}_{z}$ is:

(23) $${\hat{R}}_{z}=\frac{1}{L}\sum_{t=1}^{L}Z_{1}(t)Z_{2}^{H}(t).$$

Performing SVD on ${\hat{R}}_{z}$ can be thus expressed as:

(24) $${\hat{R}}_{z}=[{\hat{U}}_{S}\;{\hat{U}}_{N}]\left[\begin{array}{ll} {\hat{\Lambda }}_{S} & \;0\\ \;0\; & {\hat{\Lambda }}_{N} \end{array}\right]{\left[{\hat{V}}_{S}\;{\hat{V}}_{N}\right]}^{H}.$$

For small numbers of samples, the additive noise vectors does not satisfy the statistical assumptions in Equation (8). As mentioned in Ref. [40], if the number of samples is high, the singular values of the sample CCM will converge to the singular values of the CCM. On the contrary, the eigenvalues of the sample CCM will diverge in a large cluster. Therefore, ${\hat{\Lambda }}_{N}$ may not equal the null matrix for small numbers of samples.

To reduce the perturbation of additive noise in the sample CCM ${\hat{R}}_{z}$ with small numbers of samples, we make the matrix ${\hat{\Lambda }}_{N}=0$ to reconstruct the modified CCM $R_{z1}$:

(25) $$R_{z1}=[{\hat{U}}_{S}\;{\hat{U}}_{N}]\left[\begin{array}{ll} {\hat{\Lambda }}_{S} & \;0\\ \;0\; & 0 \end{array}\right]{\left[{\hat{V}}_{S}\;{\hat{V}}_{N}\right]}^{H}={\hat{U}}_{S}{\hat{\Lambda }}_{S}{\hat{V}}_{S}{}^{H}.$$

Comparing Equation (25) with Equation (24), it can be concluded that using the matrix $R_{z1}$ instead of ${\hat{R}}_{z}$ means that the sub-matrix ${\hat{U}}_{N}{\hat{\Lambda }}_{N}{\hat{V}}_{N}^{H}$ is omitted.

### 3.2. Virtual Arrays Generation Based on the CCM for Nested Arrays

In this section, we first review the virtual arrays of the original nested arrays. As mentioned in Ref. [34], the nested arrays were proposed to obtain $O(M^{2})$ DOF from M sensors by vectorizing the ACM of the received signals as follows:

(26) $$z=vec(R_{\mathrm{xx}})=(A^{*}⊙A)p+\sigma_{n}^{2}\vec{l_{n}},$$

where $R_{\mathrm{xx}}=E[X(t)X^{H}(t)]=AR_{s}A^{H}+\sigma_{n}^{2}I$ is the ACM of the received signals, X(t) is the output of the physical array, $p={\left[\sigma_{1}^{2},\sigma_{2}^{2},\ldots ,\sigma_{k}^{2}\right]}^{T}$ denotes the equivalent signal vector, $\vec{l_{n}}=[e_{1}^{T}\;e_{2}^{T}\;\ldots \;e_{N}^{T}]$ is the noise vector of the virtual arrays, $e_{i}$ is a column vector of all zeros except a 1 at the *i*-th position, and $A^{*}⊙A$ can be seen as the manifold matrix of the virtual arrays.

The locations of virtual arrays can be expressed by the set $D=\{{\vec{d}}_{i}-{\vec{d}}_{j},\;1\leq i,j\leq N\}$, where ${\vec{d}}_{i}$ denote the position vector of the *i*-th sensor. According to the analysis in Ref. [33], the set *D* contains the self-differences set $D_{s}$ and the cross-differences set $D_{c}$, defined as:

(27) $$D_{s}=\{(N_{1}+1)(k_{1}-k_{2})\}\cup \{(l_{1}-l_{2})\},1<k_{1},k_{2}<N_{2},1<l_{1},l_{2}<N_{1}$$

(28) $$D_{c}=\{(N_{1}+1)k-l\}\cup \{l-(N_{1}+1)k\},\;1<k<N_{2},1<l<N_{1},$$

where l denotes the position vector of first level subarray with spacing $d_{1}=\lambda /2$, k denotes the position vector of second level sub-array with spacing $d_{2}=(N_{1}+1)\lambda /2$, $N_{1}$ is the number of sensors in the first level subarray, and $N_{2}$ is the number of sensors in the second level subarray. From Equations (27) and (28), the cross-differences set $D_{c}$ contributes the majority of sensor locations, the self-differences set $D_{s}$ contributes the remaining sensor locations, which contain some overlapping ones. In addition, the output of the virtual arrays contains noise $\vec{l_{n}}$, which need an extra operation to remove. In order to obtain the virtual arrays without the overlapping element and noise, we propose an improved L-shaped nested arrays based on CCM, in order to take full advantage of the cross-differences property.

We divide the modified CCM $R_{z1}$ into four sub-matrices as shown in Equation (17), and then reverse the matrices $R_{x1x2}$ and $R_{y1y2}$ according to the columns, which can be expressed as:

(29) $$\begin{array}{ll} R_{x}= & \prod_{N}R_{x1x2}\\ & =\sum_{i=1}^{k}\sigma_{i}^{2}\left[\begin{array}{cccc} \;1\; & e^{jN\pi \cos\alpha_{i}} & \cdots & e^{j(N^{2}-N)\pi \cos\alpha_{i}}\\ e^{j\pi \cos\alpha_{i}} & e^{j(N+1)\pi \cos\alpha_{i}} & \cdots & e^{j(N^{2}-N+1)\pi \cos\alpha_{i}}\\ \vdots & \vdots & \vdots & \vdots \\ e^{j(N-1)\pi \cos\alpha_{i}} & \;e^{j(2N-1)\pi \cos\alpha_{i}} & \;\cdots & {\;e}^{j(N^{2}-1)\pi \cos\alpha_{i}} \end{array}\right] \end{array}$$

(30) $$\begin{array}{ll} R_{y}= & \prod_{N}R_{y1y2}\\ & =\sum_{i=1}^{k}\sigma_{i}^{2}\left[\begin{array}{cccc} \;1\; & e^{jN\pi \cos\beta_{i}} & \cdots & e^{j(N^{2}-N)\pi \cos\beta_{i}}\\ e^{j\pi \cos\beta_{i}} & e^{j(N+1)\pi \cos\beta_{i}} & \cdots & e^{j(N^{2}-N+1)\pi \cos\beta_{i}}\\ \vdots & \vdots & \vdots & \vdots \\ e^{j(N-1)\pi \cos\beta_{i}} & \;e^{j(2N-1)\pi \cos\beta_{i}} & \;\cdots & {\;e}^{j(N^{2}-1)\pi \cos\beta_{i}} \end{array}\right] \end{array}$$

We utilize the matrices $R_{x}$ and $R_{y}$ to calculate the following vectors:

(31) $$\begin{array}{ll} r_{x} & =vec(R_{x})=\mathrm{vec}(\prod_{N}A_{x1}(\alpha )R_{s}A_{x2}^{H}(\alpha ))\\ & =(A_{x2}^{*}(\alpha )⊙A_{x1z}(\alpha ))p\\ & =\bar{A_{x}}(\alpha )p \end{array}$$

(32) $$\begin{array}{ll} r_{y}= & vec(R_{y})=vec(\prod_{N}A_{y1}(\beta )R_{s}A_{y2}^{H}(\beta ))\\ & =(A_{y2}^{*}(\beta )⊙A_{y1z}(\beta ))p\\ & =\bar{A_{y}}(\beta )p \end{array}$$

where $A_{x1z}(\alpha )=\prod_{N}A_{x1}(\alpha )$, $A_{y1z}(\alpha )=\prod_{N}A_{y1}(\beta )$, and the equivalent signal vector is expressed by $p={\left[\sigma_{1}^{2},\sigma_{2}^{2},\ldots ,\sigma_{k}^{2}\right]}^{T}$. Additionally, $r_{x}$ can be seen as the output of the virtual arrays along the X-axis, whose manifold is given by $\bar{A_{x}}=[\bar{a_{x}}(\alpha_{1}),\bar{a_{x}}(\alpha_{2}),\ldots ,\bar{a_{x}}(\alpha_{k})]$; $r_{y}$ can be seen as the output of the virtual arrays along the Y-axis, whose manifold is given by $\bar{A_{y}}=[\bar{a_{y}}(\beta_{1}),\bar{a_{y}}(\beta_{2}),\ldots ,\bar{a_{y}}(\beta_{k})]$; and the *i*-th column of matrix $\bar{A_{x}}$ and $\bar{A_{y}}$ are respectively written as:

(33) $$\bar{a_{x}}(\alpha_{i})=[1,e^{j\pi \cos\alpha_{i}},\ldots ,e^{j\pi (N^{2}-1)\cos\alpha_{i}}]\in {\mathbb{C}}^{N^{2}\times 1}$$

(34) $$\bar{a_{y}}(\beta_{i})=[1,e^{j\pi \cos\beta_{i}},\ldots ,e^{j\pi (N^{2}-1)\cos\beta_{i}}]\in {\mathbb{C}}^{N^{2}\times 1}.\;$$

Defining $r_{x}={\left[\bar{x_{1}},\bar{x_{2}},\ldots ,\bar{x_{N^{2}}}\right]}^{T}$ and $r_{y}={\left[\bar{y_{1}},\bar{y_{2}},\ldots ,\bar{y_{N^{2}}}\right]}^{T}$ as the outputs of the virtual arrays, where $\bar{x_{i}}$ and $\bar{y_{i}}$ denote the output of *i*-th virtual array along the X-axis and Y-axis, respectively. The conjugate vectors of $r_{x}$ and $r_{y}$ are expressed as follows:

(35) $$\begin{array}{ll} r_{x}{}^{*} & ={\left[{\bar{x_{1}}}^{*},{\bar{x_{2}}}^{*},\ldots ,{\bar{x_{N^{2}}}}^{*}\right]}^{T}\\ & =(\bar{A_{x}}(\alpha )p{)}^{*}\\ & ={\bar{A_{x}}}^{*}(\alpha )p \end{array}$$

(36) $$\begin{array}{ll} r_{y}{}^{*} & ={\left[{\bar{y_{1}}}^{*},{\bar{y_{2}}}^{*},\ldots ,{\bar{y_{N^{2}}}}^{*}\right]}^{T}\\ & =(\bar{A_{y}}(\beta )p{)}^{*}\\ & \;={\bar{A_{y}}}^{*}(\beta )p \end{array}$$

where ${\bar{x_{i}}}^{*}$ and ${\bar{y_{i}}}^{*}$ denote the conjugate vector of the *i*-th virtual array’s output along the X-axis and Y-axis, $p^{*}=p={\left[\sigma_{1}^{2},\sigma_{2}^{2},\ldots ,\sigma_{k}^{2}\right]}^{T}$, ${\bar{A_{x}}}^{*}=[{\bar{a_{x}}}^{*}(\alpha_{1}),{\bar{a_{x}}}^{*}(\alpha_{2}),\ldots ,{\bar{a_{x}}}^{*}(\alpha_{k})]$, and ${\bar{A_{y}}}^{*}=[{\bar{a_{y}}}^{*}(\beta_{1}),{\bar{a_{y}}}^{*}(\beta_{2}),\ldots ,{\bar{a_{y}}}^{*}(\beta_{k})]$. The *i*-th column of the matrices of ${\bar{A_{x}}}^{*}$ and ${\bar{A_{y}}}^{*}$ are written as:

(37) $${\bar{a_{x}}}^{*}(\alpha_{i})=[1,e^{-j\pi \cos\alpha_{i}},\ldots ,e^{-j(N^{2}-1)\pi \cos\alpha_{i}}]\in {\mathbb{C}}^{N^{2}\times 1}$$

(38) $${\bar{a_{y}}}^{*}(\beta_{i})=[1,e^{-j\pi \cos\beta_{i}},\ldots ,e^{-j\pi (N^{2}-1)\cos\beta_{i}}]\in {\mathbb{C}}^{N^{2}\times 1}.$$

According to the Equations (35) and (36), $r_{x}{}^{*}$ and $r_{y}{}^{*}$ can be seen as the outputs of virtual arrays whose manifold are given by ${\bar{A_{x}}}^{*}$ and ${\bar{A_{y}}}^{*}$, respectively. Defining $\bar{X}={\left[{\bar{x_{N^{2}}}}^{*},\ldots ,{\bar{x_{2}}}^{*},\bar{x_{1}},\ldots ,\bar{x_{N^{2}}}\right]}^{T}$, which behaves like the output of a uniform linear array along the X-axis containing $2N^{2}-1$ elements with spacing $d_{1}=\lambda /2$, and $\bar{Y}={\left[{\bar{y_{N^{2}}}}^{*},\ldots ,{\bar{y_{2}}}^{*},\bar{y_{1}},\ldots ,\bar{y_{N^{2}}}\right]}^{T}$, which behaves like the output of a uniform linear array along the Y-axis containing $2N^{2}-1$ elements with spacing $d_{1}=\lambda /2$. Thus, we obtain the virtual arrays of $4N^{2}-2$ elements in the X-Y plane using only 4N−2 physical sensors, which dramatically increases the DOF.

### 3.3. Improved Unitary ESPRIT Algorithm for Received Signals of Virtual Arrays

According to the outputs of the virtual arrays in Equations (31), (32), (35), and (36), the equivalent signal vector p behaves like coherent signal sources that lead to a rank deficiency of the covariance matrix. Hence, the conventional high-resolution DOA estimation algorithm such as MUSIC or ESPRIT can’t be directly used for the virtual arrays. In this subsection, the equivalent covariance matrix is constructed to estimate the DOA of the virtual arrays.

Since the covariance matrix is a Toeplitz matrix, we can use $\bar{X}$ to construct the equivalent covariance matrix $R_{X}$:

(39) $$R_{X}=\left[\begin{array}{lllll} \bar{x_{1}} & \bar{x_{2}{}^{*}} & \bar{x_{3}{}^{*}} & \ldots & \bar{x_{N^{2}}{}^{*}}\\ \bar{x_{2}} & \bar{x_{1}} & \bar{x_{2}{}^{*}} & \ldots & \bar{x_{N^{2}-1}{}^{*}}\\ \vdots & \vdots & \vdots & \vdots & \vdots \\ \bar{x_{N^{2}}} & \bar{x_{N^{2}-1}} & \bar{x_{N^{2}-2}} & \ldots & \bar{x_{1}} \end{array}\right]\;=\bar{A_{x}}\left(\begin{array}{ccc} \sigma_{1}^{2} & \ldots & 0\\ \vdots & \ddots & \vdots \\ 0 & \cdots & \sigma_{k}^{2} \end{array}\right){\bar{A_{x}}}^{H}.$$

From Equation (33), $\bar{A_{x}}$ is Vandermonde matrix that satisfies the property of rotational invariance. After performing the EVD of the matrix $R_{X}$, the rotational invariance property can be expressed as:

(40) $$J_{1}U_{s}\Phi_{x}=J_{2}U_{s},$$

where $U_{s}$ is the matrix composed of eigenvector corresponding to the *K* largest eigenvalues, $J_{1}=[I_{N^{2}-1},0_{(N^{2}-1)\times 1}]$, $J_{2}=[0_{(N^{2}-1)\times 1},I_{N^{2}-1}]$, and $\Phi_{x}=diag[e^{j\pi \cos\alpha_{1}},e^{j\pi \cos\alpha_{2}},\ldots ,e^{j\pi \cos\alpha_{k}}]$.

The ESPRIT algorithm needs to perform SVD or EVD on the complex covariance matrix which means leads to a high computational burden. To reduce the computational complexity of the algorithm, the unitary transformation is used to transform the complex covariance matrix into a real-valued matrix [41]. We construct the Centro-Hermitian matrix $R_{X1}$:

(41) $$R_{X1}=\frac{1}{2}(R_{X}+\prod_{N^{2}}R_{X}^{*}\prod_{N^{2}}).$$

Additionally, we define the unitary matrix:

(42) $$Q_{n}=\left\{ \begin{array}{ll} \frac{1}{\sqrt{2}}\left[\begin{array}{ll} I_{m} & \;jI_{m}\\ \prod_{m} & \;-j\prod_{m} \end{array}\right] & n=2m\;\\ \frac{1}{\sqrt{2}}\left[\begin{array}{lll} I_{m} & 0_{\left(m\times 1\right)} & jI_{m}\\ 0_{\left(1\times m\right)} & \sqrt{2} & 0_{\left(1\times m\right)}\\ \prod_{m} & 0_{\left(m\times 1\right)} & -j\prod_{m} \end{array}\right] & n=2m+1\; \end{array}\right..$$

We use the unitary matrix $Q_{n}$ to transform the complex matrix $R_{X1}$ into real matrix $R_{X2}$ via:

(43) RX2=12QN2H(RX+∏N2RX*∏N2)QN2=12(QN2HRXQN2+QN2H∏N2RX*∏N2QN2)=12(QN2HRXQN2+QN2TRX*QN2*)=Re(QN2HRXQN2)

After the unitary transformation, the complex rotational invariance property of Equation (40) becomes the real-valued rotation; invariance property as follows:

(44) $$K_{1}E_{sx}\Psi_{x}=K_{2}E_{sx},$$

where $K_{1}=Q_{N^{2}-1}^{H}(J_{1}+J_{2})Q_{N^{2}}$, $K_{2}=Q_{N^{2}-1}^{H}j(J_{1}-J_{2})Q_{N^{2}}$, and $\Psi_{x}=diag[\tan(\pi \cos\alpha_{1}/2),\ldots ,\tan(\pi \cos\alpha_{k}/2)]$.

From the Equation (44), the $\Psi_{x}$ can be obtained by the least square method:

(45) $$\Psi_{x}=(K_{1}E_{sx}{)}^{+}K_{2}E_{sx}.$$

By performing EVD on $\Psi_{x}$, the angle $\hat{\alpha_{i}}$ can be obtained from the *i*-th eigenvalue $a_{i}$ of $\Psi_{x}$ as follow:

(46) $$\hat{\alpha_{i}}=\arccos(2\arctan(a_{i})/\pi ).$$

Similarly, we use $r_{y}$ and $r_{y}{}^{*}$ to construct the equivalent covariance matrix $R_{Y}$:

(47) $$R_{Y}=\left[\begin{array}{lllll} \bar{y_{1}} & \bar{y_{2}{}^{*}} & \bar{y_{3}{}^{*}} & \ldots & \bar{y_{N^{2}}{}^{*}}\\ \bar{y_{2}} & \bar{y_{1}} & \bar{y_{2}{}^{*}} & \ldots & \bar{y_{N^{2}-1}{}^{*}}\\ \vdots & \vdots & \vdots & \vdots & \vdots \\ \bar{y_{N^{2}}} & \bar{y_{N^{2}-1}} & \bar{y_{N^{2}-2}} & \ldots & \bar{y_{1}}\; \end{array}\right]=\bar{A_{y}}\left(\begin{array}{ccc} \sigma_{1}^{2} & \ldots & 0\\ \vdots & \ddots & \vdots \\ 0 & \cdots & \sigma_{k}^{2} \end{array}\right){\bar{A_{y}}}^{H}.$$

We then use the unitary matrix $Q_{n}$ to transform $R_{Y}$ into real matrix $R_{Y1}$ as follow:

(48) RY1=12QN2H(RY+∏N2RY*∏N2)QN2=Re(QN2HRYQN2)

After the unitary transformation, the real-valued rotation invariant property is written as:

(49) $$K_{1}E_{sy}\Psi_{y}=K_{2}E_{sy}$$

where $\Psi_{y}=diag[\tan(\pi \cos\beta_{1}/2),\ldots ,\tan(\pi \cos\beta_{k}/2)]$.

Then $\Psi_{y}$ can be obtained by the least square method. After performing EVD on $\Psi_{y}$, the angle ${\hat{\beta }}_{i}$ can be obtained from the *i*-th eigenvalue $b_{i}$ of the matrix $\Psi_{y}$ as follows:

(50) $$\Psi_{y}=(K_{1}E_{sy}{)}^{+}K_{2}E_{sy}$$

(51) $$\hat{\beta_{i}}=\arccos(2\arctan(b_{i})/\pi ).$$

### 3.4. Pair Matching

Since the angles $\hat{\alpha }$ and $\hat{\beta }$ are separately obtained by 1-D DOA estimation. This may lead to a mismatch between angle $\hat{\alpha }$ and angle $\hat{\beta }$ with more than one incident signal. From Equations (31) and (32), the equivalent signal vector p of different virtual arrays is the same vector. The manifold matrix of virtual arrays $\bar{A_{x}}(\hat{\alpha })$ can be calculated with the estimated angle $\hat{\alpha }$. From Equation (31), the equivalent signal vector $p_{1}$ can be derived by the least square method as follows:

(52) $${\hat{p}}_{1}=\arg\underset{p}{\min}{\left\|r_{x}-\bar{A_{x}}(\hat{\alpha }){\hat{p}}_{1}\right\|}^{2}$$

(53) $${\hat{p}}_{1}={\left[{\bar{A_{x}}}^{H}(\hat{\alpha })\bar{A_{x}}(\hat{\alpha })\right]}^{-1}{\bar{A_{x}}}^{H}(\hat{\alpha })r_{x}$$

Similarly, the manifold matirx $\bar{A_{y}}(\beta )$ can be calculated with the estimated angle $\hat{\beta }$. From Equation (32), the equivalent signal vector $p_{2}$ can be derived via:

(54) $${\hat{p}}_{2}=\arg\underset{p}{\min}{\left\|r_{y}-\bar{A_{y}}(\hat{\beta }){\hat{p}}_{2}\right\|}^{2}$$

(55) $${\hat{p}}_{2}={\left[{\bar{A_{y}}}^{H}(\hat{\beta })\bar{A_{y}}(\hat{\beta })\right]}^{-1}{\bar{A_{y}}}^{H}(\hat{\beta })r_{y}.$$

The elements of the equivalent signal vector $p={\left[\sigma_{1}^{2},\sigma_{2}^{2},\ldots ,\sigma_{k}^{2}\right]}^{T}$ denote the powers of the incident signals. Due to the different power of incident signals, the pair matching problem can be done by sorting the elements of ${\hat{p}}_{1}$ and ${\hat{p}}_{2}$:

(56) $$∐=\arg\underset{∐}{\min}\left\|{\hat{p}}_{1}-∐{\hat{p}}_{2}\right\|,$$

where the ∐ is the sorting matrix. Then the result of pair matching is the following:

(57) $$\hat{\alpha }=∐\hat{\beta }.$$

According to Equations (53) and (55), the powers of incident signals have also been estimated.

### 3.5. Algorithm Implementation and Complexity Analysis

The proposed algorithm can be summarized as follows:

Step 1: Divide the nested arrays of into four subarrays. Construct the vectors $Z_{1}(t)$ and $Z_{2}(t)$ with the outputs of the subarrays. Then calculate the CCM $R_{z}=E[Z_{1}(t)Z_{2}^{H}(t)]$.

Step 2: Perform SVD on the matrix $R_{z}$ to reconstruct the modified CCM $R_{z1}$ via Equation (25).

Step 3: Divide the modified CCM $R_{z1}$ into four sub-matrices as in Equation (17), then use the submatrices $R_{x1x2}$ and $R_{y1y2}$ to obtain the output of the virtual arrays via Equations (29)–(32).

Step 4: Construct the equivalent covariance matrices $R_{X}$ and $R_{Y}$ with the output of the virtual arrays via Equations (39) and (47).

Step 5: Utilize the unitary matrix to transform the covariance matrix into a real-valued covariance matrix. Then conduct the ESPRIT algorithm on the real-valued covariance matrix to estimate the angles $\hat{\alpha }$ and $\hat{\beta }$.

Step 6: Derive the equivalent source signal vectors $p_{1}$ and $p_{2}$ with the estimated angles $\hat{\alpha }$ and $\hat{\beta }$ via Equations (52) and (54), respectively. Derive the sorting matrix ∐, and pair the angles $\hat{\alpha }$ and $\hat{\beta }$ by using the sorting matrix ∐.

As for the complexity of algorithm, the main computation of algorithm contains the reconstructing CCM, SVD operation, pseudo-inverse operation and the least square method. Considering that the virtual arrays extend the dimension of the equivalent covariance matrix, which need a lot of calculations, the unitary transformation is performed on equivalent covariance matrix to reduce the computational complexity of the proposed algorithm. The main computational complexity of our proposed algorithm is $O[(M+1{)}^{2}L+{\left(M+1\right)}^{3}+{\left(M+1\right)}^{6}/128+5{\left(M+1\right)}^{2}k^{2}/8+{\left(M+1\right)}^{2}k/8+k^{3}/2]$, where M denotes the number of sensors, L denotes the number of snapshots, k denotes the number of incident signals. The algorithm of joint singular value decomposition (JSVD) [24] involves a beamforming-like spectral search. By defining the search step of 0.1 degree, the computational complexity of JSVD is about $O[M^{2}L+8M^{3}+1800M^{2}]$. On the other hand, the method of cross-correlation matrices propagator method (CCMs-PM) [29], which obtain the DOAs without EVD or SVD, cost approximately $O[M^{2}L+2k^{3}+(7M-4)k^{2}+k(M-1)(2M-k)]$. The methods of JSVD and CCMs-PM are applied on L-shaped uniform arrays. The method of signal subspace joint diagonalization (SSJD) [39], performed on nested arrays, requires a complexity of $O[3M^{2}L(2N-1)+4{\left((M^{2}-1)/2+M^{2}+M\right)}^{2}(2N-1)+8{\left((M^{2}-1)/2+M^{2}+M\right)}^{3}+10k^{3}]$, where the *N* is frame number of the method. For the sake of clarity, the main complexity of the proposed algorithm, the JSVD algorithm, the SSJD algorithm, and the CCMs-PM algorithm are listed in Table 1.

**Figure 3:**
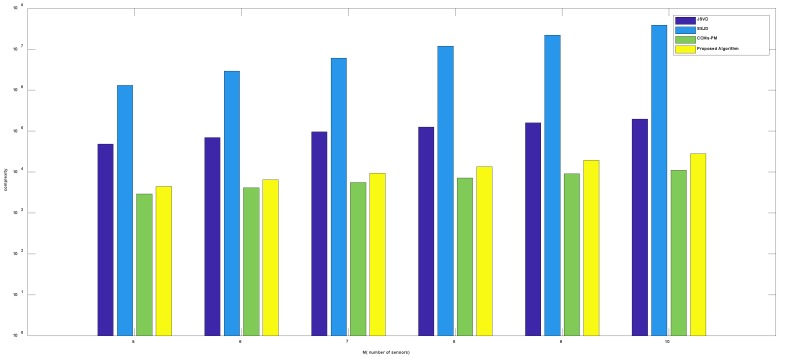
Comparison of complexity versus the number of sensors.

**Figure 4:**
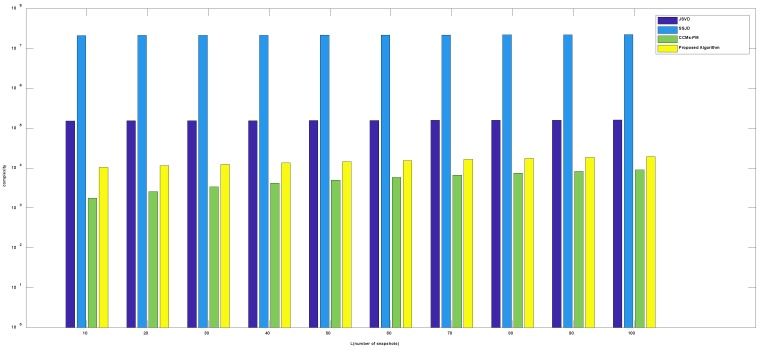
Comparison of complexity versus the number of snapshots.

Figure 3 shows the complexity comparison of algorithms versus the number of sensors, where the number of snapshots *L* is 100, the number of incident signals *k* is 3. Figure 4 shows the complexity comparison of algorithms versus the number of snapshots, where the number of sensors *M* is 9, the number of incident signals *k* is 3. From Figure 3 and Figure 4, the complexity of the proposed algorithm is much lower than the JSVD and SSJD algorithms. The low computational complexity is obtained from the unitary transformation of the complex matrix into a real-valued matrix. Although the complexity of the proposed algorithm is a bit higher than the CCMs-PM, the computational complexity of the proposed algorithm is basically on the same order as the complexity of CCMs-PM.

## 4. Simulation Results and Performance Analysis

In this section, several simulation experiments are conducted to verify the performance of the proposed algorithm. Consider L-shaped nested arrays consisting of two orthogonal nested arrays, where each nested array contains nine sensors, of which the inner ULA contains five sensors with an inter-sensor spacing of λ/2 and the outer ULA contains four sensors with an inter-sensor spacing 5λ/2, where λ is the wavelength of the incident waves. Consider that three signals are impinging from the locations $\left(\alpha_{1},\beta_{1})=(25^{\circ },30^{\circ }\right)$, $\left(\alpha_{2},\beta_{2})=(35^{\circ },40^{\circ }\right)$, and $\left(\alpha_{3},\beta_{3})=(45^{\circ },50^{\circ }\right)$. The power of signals are set as $\sigma_{1}^{2}=3$, $\sigma_{2}^{2}=4$, and $\sigma_{3}^{2}=5$. The input signal-to-noise-ratio (SNR) is defined as $SNR=10\log_{10}(\sigma_{s}{}^{2}/\sigma_{n}{}^{2})$, where $\sigma_{s}{}^{2}$ and $\sigma_{n}{}^{2}$ denote the power of signal and noise, respectively.

Two criteria for assessing the performance of the DOA estimation algorithm are the probability of resolution and the root mean square error (RMSE). The probability of resolution is defined as the probability that the angle difference between the estimated angle and the real angle is less than half of the beam width among several Monte-Carlo experiments, which can be expressed as follows:

(58) $$P=P\left(\max\left\{\left|{\hat{\alpha }}_{i,j}-\alpha_{j}\right|,\left|{\hat{\beta }}_{i,j}-\beta_{j}\right|\right\}\leq \frac{BW}{2}\right)$$

where BW denotes the beam width.

The definition of RMSE is as follows:

(59) $$RMSE=\sqrt{\frac{1}{nk}\sum_{i=1}^{n}\sum_{j=1}^{k}({\left({\hat{\alpha }}_{i,j}-\alpha_{j}\right)}^{2}+({\hat{\beta }}_{i,j}-\beta_{j}{)}^{2})}$$

where ${\hat{\alpha }}_{i,j}$ and ${\hat{\beta }}_{i,j}$ are the estimated angles of *j*-th incident signal for *i*-th Monte-Carlo experiment. Likewise, $\alpha_{j}$ and $\beta_{j}$ are the true angles of *j*-th incident signal. Additionally, *n* is the number of Monte-Carlo trials, and *k* denotes the number of incident signals.

**Figure 5:**
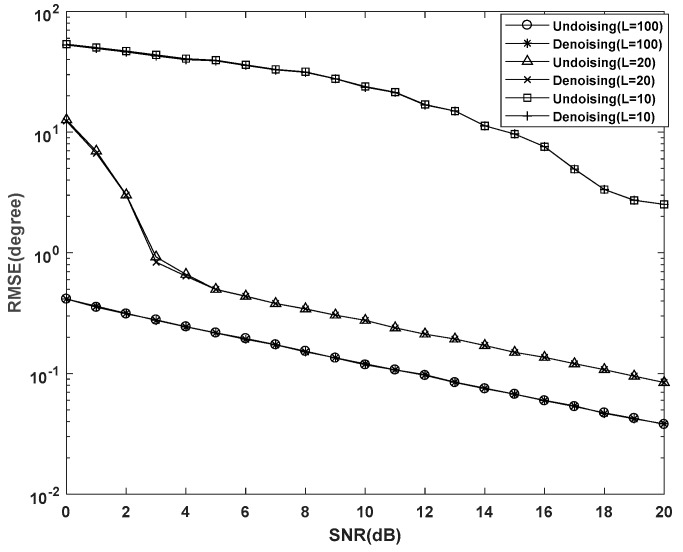
Effects of omitting the noise on root mean square error (RMSE) performance.

In first experiment, we examine the effect of the noise term $\sigma_{n}{}^{2}R_{n0}$ in Equations (18) and (21) on DOA estimation. We compare the RMSE of the proposed algorithm with the denoising algorithm, which removed the noise term $\sigma_{n}{}^{2}R_{n0}$ from the CCM and the other steps are the same as the proposed algorithm. The RMSE of the proposed algorithm and denoising algorithm versus SNR under 1000 Monte-Carlo simulations is shown in Figure 5, where the number of snapshots *L* is 10, 20 and 100, respectively. From Figure 5, the RMSE of the proposed algorithm and denoising algorithm under different numbers of snapshots are almost overlapping in all cases, which verifies the rationality of omitting the noise term in Equations (18) and (21).

**Figure 6:**
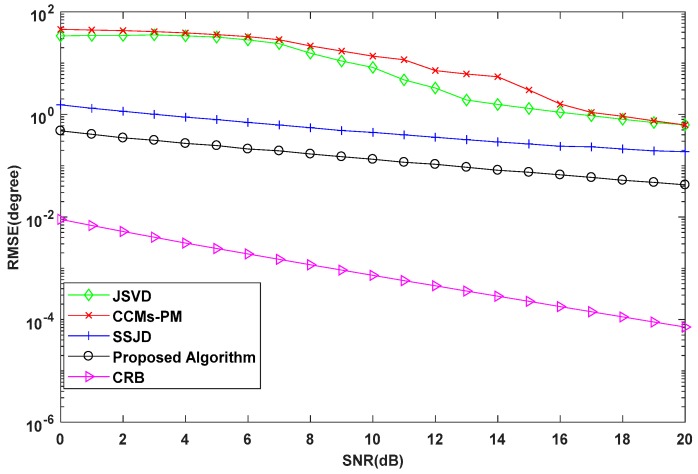
RMSE performance versus signal-to-noise-ratio (SNR) for simulations with 100 snapshots.

**Figure 7:**
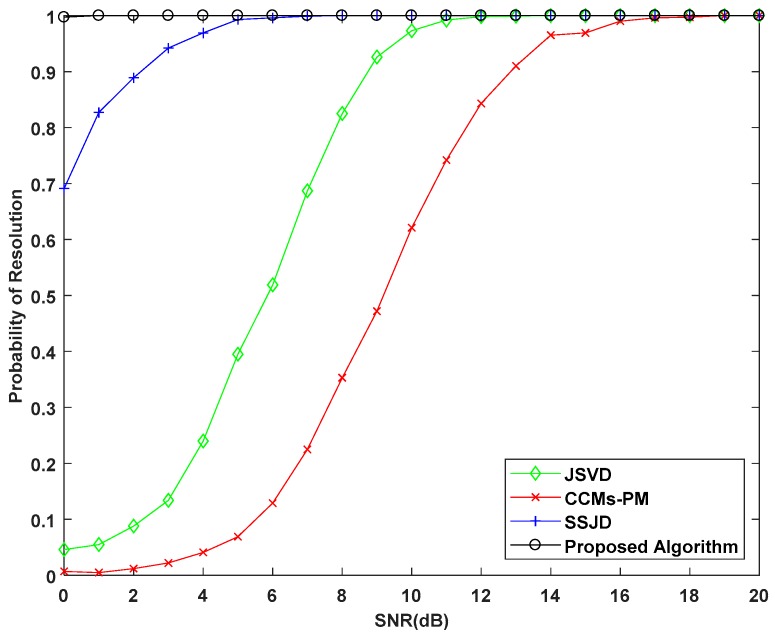
Probability of Resolution versus SNR for simulation with 100 snapshots.

In the second experiment, we examine the DOA estimation performance of the proposed algorithm versus the SNR in terms of the root mean square error (RMSE). We also compare the proposed algorithm with the CRB, and the JSVD [24], CCMs-PM [29], SSJD algorithms [39]. The JSVD and CCMs-PM algorithms are performed on L-shaped uniform arrays, whose sensor spacing is λ/2. The SSJD algorithm is performed on the L-shaped nested arrays. The CRB of L-shaped nested arrays is derived in Appendix A. Figure 6 and Figure 7 show the RMSE and the probability of resolution of the different algorithms versus the SNR for 1000 Monte-Carlo simulations, where the number of snapshots *L* is 100.

As shown in Figure 6, the RMSE of the proposed algorithm is lower than other compared algorithms for all SNRs. From Figure 7, we see the probability of resolution for all algorithms is improved with the increase of SNR, and the probability of resolution for the proposed algorithm is constant equal to 100%, which is obviously better than other compared algorithms. These results confirm that our proposed algorithm outperforms the JSVD algorithm and CCMs-PM algorithm due to the larger DOF obtained from nested arrays. Moreover, the computational complexity of the proposed algorithm is not higher than the JSVD algorithm and CCMs-PM algorithm. Compared with the SSJD algorithm, our algorithm also has better angle estimation performance by utilizing the reconstructed CCM instead of the ACM, which removes the noise vector.

In the third experiment, we evaluate the DOA estimation performance of the proposed algorithm versus the number of snapshots. The SNR is set to 10 dB and 20 dB. The number of snapshots varies from 20 to 200. Similarly, we compare the performance of the proposed algorithm with CRB, and the JSVD, CCMs-PM, and SSJD algorithms with 1000 Monte Carlo trials.

**Figure 8:**
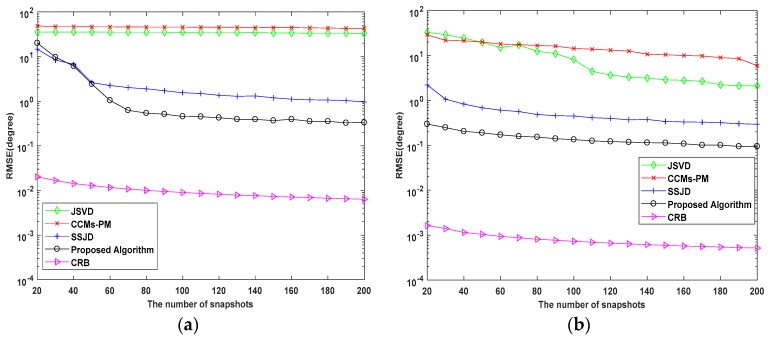
RMSE performance versus the number of snapshots for (**a**) SNR = 0 dB; (**b**) SNR = 10 dB.

**Figure 9:**
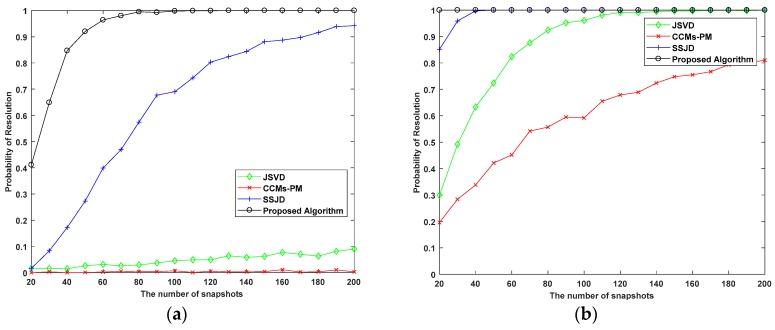
Probability of Resolution versus snapshots (**a**) SNR = 0 dB; (**b**) SNR = 10 dB.

As shown in Figure 8 and Figure 9, the RMSE and the probability of resolution depicts that the performance of the proposed algorithm still outperforms the contrasting algorithms for the studied numbers of snapshots. Since the JSVD and CCMs-PM are vulnerable to noise, the performance of these algorithms seriously deteriorates under a low SNR environment. From Figure 8 and Figure 9, when SNR = 0 dB and the number of snapshots is less than 60, the RMSE of the proposed algorithm decreases rapidly with the increasing of snapshots, which is closed to the RMSE of SSJD. When the SNR is 0 dB and the number of snapshots is more than 60, the RMSE of the proposed algorithm decreases smoothly with the increasing number of snapshots, and the probability of resolution of the proposed algorithm is above 90%. When SNR = 10 dB, the RMSE of the proposed algorithm decrease smoothly with the increasing number of snapshots, and when the number of snapshots is more than 60, the proposed algorithm’s probability of resolution is almost 100%. Figure 8 and Figure 9 indicate that our proposed algorithm has a better performance under the conditions of low SNR and a small number of snapshots.

**Figure 10:**
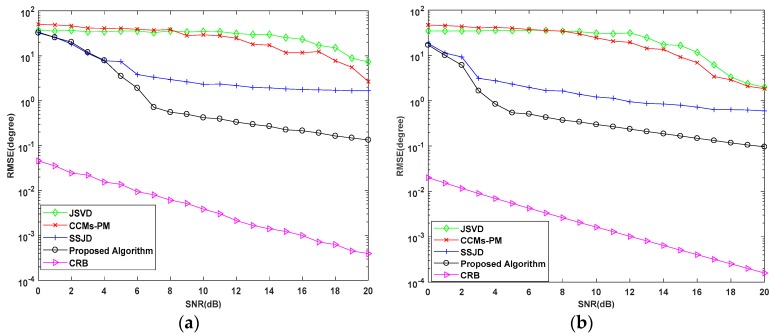
RMSE performance versus SNR for a small number of snapshots with (**a**) snapshots = 10; (**b**) snapshots = 20.

**Figure 11:**
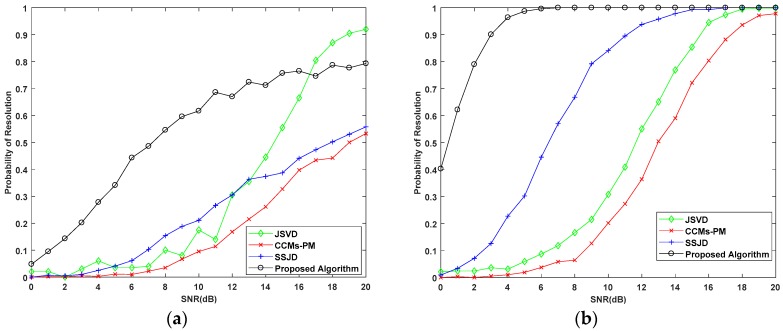
Probability of Resolution versus SNR for a small number of snapshots with (**a**) snapshots = 10; (**b**) snapshots = 20.

In the fourth experiment, we examine the estimation performance of the proposed algorithm versus SNR for a small number of snapshots, where the SNR varies from 0 dB to 20 dB, and the number of snapshots are 10 and 20. Similarly, we compare the performance of the proposed algorithm with the CRB, and the JSVD, CCMs-PM, and SSJD algorithms with 1000 Monte Carlo trials. Figure 10 and Figure 11 show the performance of the above algorithms versus SNR for a small number of snapshots.

As shown in Figure 10, the proposed algorithm can obtain better estimation performance than the other algorithms. From Figure 10, the RMSE of the JSVD and CCMs-PM algorithms decline slowly at low SNR for a small number of snapshots. These results show that the effect of removing noise through the CCM is sensitive to the number of snapshots. The performance of SSJD is better than the JSVD and CCMs-PM algorithms, but inferior to the proposed algorithm. With an increase in the SNR, the estimation performance of the proposed algorithm dramatic decreased compared to the above algorithms, for a small number of snapshots. This is due to the reconstruction of the CCM based on SVD. When the SNR is greater than 10 dB, the RMSE of our algorithm is less than 1 degree with 10 snapshots, which verifies the effectiveness of the proposed algorithm under a small number of snapshots.

From Figure 11, when the number of snapshots is 10 and SNR is lower than 15 dB, the probability of the proposed algorithm is higher than other algorithms. When the number of snapshots is 10 and the SNR is higher than 15 dB, the probability of the JSVD algorithm, performed on the uniform arrays, is better than the proposed algorithm. The beam width is proportional to the array aperture. Therefore, the beam width for uniform L-shaped arrays is much higher than the nested L-shaped arrays, which make the probability of JSVD higher than the proposed algorithm in a high SNR environment. When the number of snapshots is 20, the probability of resolution for proposed algorithm is higher than the other compared algorithms for all studied SNRs. Furthermore, when SNR is higher than 10 dB, the probability of the proposed algorithm is 100%, which denotes the optimal performance of our algorithm.

For the sake of clarity, the RMSE of the proposed algorithm, the JSVD, the SSJD, and the CCMs-PM algorithms are listed in Table 2, where the number of snapshots are 10 and 20, the SNR are 0 dB, 5 dB, 10 dB.

As described in Table 2, the RMSE of the proposed algorithm is obviously lower than other compared algorithms, which indicates the best estimation performance among all considered algorithms for a small number of samples.

In the last experiment, the estimation performance of the proposed algorithm versus SNR for different sensor spacing. The SNR varies from 0 dB to 20 dB, and the number of snapshots is 20. The sensor spacing of inner ULA of nested arrays for simulation are 0.5λ and 0.4λ, respectively. The sensor spacing of outer ULA of nested arrays for simulation are 2.5λ and 2λ, respectively. Similarly, the sensor spacing of L-shaped uniform arrays for simulation are 0.5λ and 0.4λ, respectively. For ease of expression in the following analysis, we define the symbol *d* as the sensor spacing of L-shaped uniform arrays and the sensor spacing of inner ULA of nested arrays. We compare the performance of the proposed algorithm with Cramér–Rao bound (CRB), JSVD, CCMs-PM, and SSJD algorithms with 1000 Monte Carlo trials.

**Figure 12:**
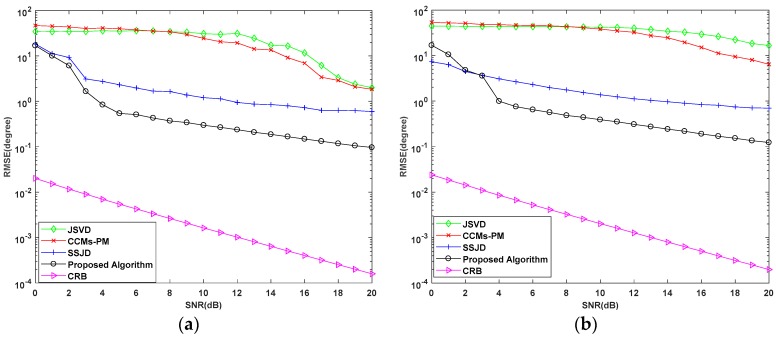
RMSE Performance versus SNR for different sensor spacing (**a**) d=0.5λ; (**b**) d=0.4λ.

**Figure 13:**
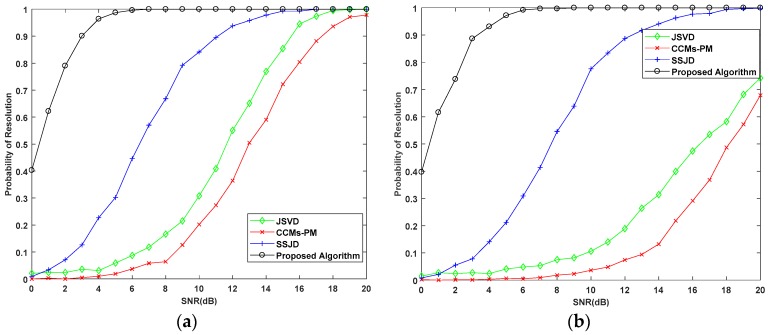
Probability of Resolution versus SNR for different sensor spacing (**a**) d=0.5λ; (**b**) d=0.4λ.

As shown in Figure 12, as the increasing of SNR, the RMSE of the proposed algorithm is the lower than the other algorithms for different sensor spacing, which indicates the best performance of the proposed algorithm. From Figure 13, the probability of resolution of proposed algorithm is higher than the compared algorithms. When SNR is higher than 5 dB, the RMSE of the proposed algorithm is lower than 1 degree and the probability of resolution is almost 100% with both of the sensor spacing. Compare the RMSE curves and the probability of resolution curves with different sensor spacing, the RMSE for all of the algorithms with sensor spacing d=0.5λ are lower than these with sensor spacing d=0.4λ. The probability of resolution with sensor spacing d=0.5λ of all the algorithms is higher than these with sensor spacing d=0.4λ. This indicates that the estimation performance with bigger sensor spacing is better than the one with little sensor spacing. This is because that the sensor spacing is proportional to the effective array aperture, which determines the estimation accuracy of the estimation. However, the DOA estimation will lead to angle ambiguity when the sensor spacing of *d* is bigger than 0.5λ. Therefore, we choose the sensor spacing d=λ/2 as the array configuration of array sensor.

## 5. Conclusions

In this paper, we have presented an improved 2-D DOA estimation algorithm using an L-shaped nested array. To improve the estimation of CCM under small numbers of samples, a new CCM based on SVD was constructed. A novel virtual generation of nested array is considered by utilizing the reconstructed CCM without the overlapping element and noise vector. After obtaining the virtual arrays, an improved unitary ESPRIT algorithm is performed on the full rank equivalent variance matrices, which are constructed by the output of the virtual arrays and their conjugate vectors, to estimate the azimuth, and elevation angles. The pair-matching method is presented by estimating the equivalent signal vector of the virtual arrays, which also estimate the power of incident signals. Several numerical experiments show a better estimation performance of the proposed algorithm in contrast to others, especially under a small number of snapshots. Considering the outperformance of the proposed algorithm under small samples, our algorithm can be directly used on mono-pulse angle measurement for radar and close-range directional detection.

## Figures and Tables

**Table 1:** Comparison of the computational complexity for different algorithms.

Algorithm	Main Computational Complexity
Proposed algorithm	$O[(M+1{)}^{2}L+{\left(M+1\right)}^{3}+{\left(M+1\right)}^{6}/128+5{\left(M+1\right)}^{2}k^{2}/8+{\left(M+1\right)}^{2}k/8+k^{3}/2]$
JSVD SSJD CCMs-PM	$O[M^{2}L+8M^{3}+1800M^{2}]$ $O[3M^{2}L(2N-1)+4{\left((M^{2}-1)/2+M^{2}+M\right)}^{2}(2N-1)+8{\left((M^{2}-1)/2+M^{2}+M\right)}^{3}+10k^{3}]$ $O[M^{2}L+2k^{3}+(7M-4)k^{2}+k(M-1)(2M-k)]$

**Table 2:** Comparison of estimation RMSE for different algorithms.

Algorithm	Snapshots	RMSE (0 dB)	RMSE (5 dB)	RMSE (10 dB)
JSVD	10	36.63°	35.52°	34.70°
CCMs-PM	10	49.84°	40.54°	29.28°
SSJD	10	33.44°	7.40°	2.33°
Proposed Algorithm	10	32.40°	3.52°	0.42°
JSVD	20	34.63°	35.12°	30.88°
CCMs-PM	20	46.68°	39.79°	24.43°
SSJD	20	18.28°	2.32°	1.21°
Proposed Algorithm	20	16.93°	0.5°	0.30°

## Abbreviations

DOA	direction of arrival
CRB	Cram’er-Rao boundary
JSVD	joint singular value decomposition
ACM	autocorrelation matrix
CCM	cross correlation matrix
ESPRIT	estimation of signal parameters via rotational invariance technique
EVD	eigenvalue decomposition
PM	propagator method
ULA	uniform linear array
SLA	sparse linear arrays
SSJD	signal subspace joint diagonalization technique
SNR	signal-to-noise-ratio
SVD	singular value decomposition

## Appendix A. CRB Analysis of L-Shaped Nested Arrays

The celebrated CRB expression which offers a theoretical lower bound on the unbiased DOA estimation for uniform arrays is not suitable for nested arrays. According to Refs. [42,43], we derive the CRB expression for L-shaped nested arrays.

From Equations (2) and (3), the combined received signal Z(t) is defined as:

(A1) $$Z(t)=\left[\begin{array}{l} X(t)\\ Y(t) \end{array}\right]=\left[\begin{array}{l} A_{x}(\alpha )\\ A_{y}(\beta ) \end{array}\right]S(t)+\left[\begin{array}{l} N_{x}(t)\\ N_{y}(t) \end{array}\right]=AS(t)+N(t),$$

where $A={\left[A_{x}^{T}(\alpha )\;,A_{y}^{T}(\beta )\right]}^{T}$, and $N(t)={\left[N_{x}^{T}(t)\;,N_{y}^{T}(t)\right]}^{T}$.

The covariance matrix is calculated via:

(A2) $$R=E[Z(t)Z^{H}(t)]=AR_{s}A^{H}+\sigma^{2}I$$

where $R_{s}=S(t)S^{H}(t)/N=\mathrm{diag}(p_{1},p_{2},\ldots ,p_{k})$ denotes the source covariance matrix. Furthermore, define η as the parameter vector:

(A3) $$\eta =[\alpha_{1},\ldots ,\alpha_{k},\beta_{1},\ldots ,\beta_{k},p_{1},p_{2},\ldots ,p_{k},\sigma^{2}{]}^{T}.$$

The (m,n)-th element of Fisher Information Matrix (FIM) is given by

(A4) $$\begin{array}{ll} F_{\left(m,n\right)} & =Ntr\left(R^{-1}\frac{\partial R}{\partial \eta_{m}}R^{-1}\frac{\partial R}{\partial \eta_{n}}\right)\\ & =N{\left[vec\left(\frac{\partial R}{\partial \eta_{m}}\right)\right]}^{H}{\left(R^{T}⊗R\right)}^{-1}vec\left(\frac{\partial R}{\partial \eta_{n}}\right)\\ & =N{\left(\frac{\partial r}{\partial \eta_{m}}\right)}^{H}{\left(R^{T}⊗R\right)}^{-1}\left(\frac{\partial r}{\partial \eta_{n}}\right) \end{array}$$

where $r=vec(R)=(A^{*}⊙A)p+\sigma^{2}l_{n}=\bar{A}p+\sigma^{2}l_{n}$, and $p={\left[p_{1},p_{2},\ldots ,p_{k}\right]}^{T}$.

From Equations (A2) and (A3),

(A5) $$\begin{array}{ll} \frac{\partial r}{\partial \eta } & =\left[\frac{\partial r}{\partial \alpha_{1}},\ldots ,\frac{\partial r}{\partial \alpha_{k}},\frac{\partial r}{\partial \beta_{1}},\ldots ,\frac{\partial r}{\partial \beta_{k}},\frac{\partial r}{\partial p_{1}},\ldots ,\frac{\partial r}{\partial p_{k}},\frac{\partial r}{\partial \sigma^{2}}\right]\\ & =\left[\left(\frac{\partial A^{*}}{\partial \alpha }⊙A+A^{*}⊙\frac{\partial A}{\partial \alpha }\right)P,\left(\frac{\partial A^{*}}{\partial \beta }⊙A+A^{*}⊙\frac{\partial A}{\partial \beta }\right)P,\left(A^{*}⊙A\right),l_{n}\right] \end{array}$$

where $P=\mathrm{diag}(p)=diag[p_{1},p_{2},\ldots ,p_{k}]$,$\theta =\left[\alpha_{1},\ldots ,\alpha_{k},\beta_{1},\ldots ,\beta_{k}\right]$.

According to Equation (A4), the FIM can be expressed as:

(A6) $$F=N\left[\begin{array}{l} M_{\theta }^{H}M_{\theta }\;\;M_{\theta }^{H}M_{s}\\ M_{s}^{H}M_{\theta }\;\;M_{s}^{H}M_{s} \end{array}\right]$$

where $M_{\theta }={\left(R^{T}⊗R\right)}^{-1/2}\frac{\partial \bar{A}}{\partial \theta }P$, and $M_{s}={\left(R^{T}⊗R\right)}^{-1/2}\left[\left(A^{*}⊙A\right),l_{n}\right]$. Thus, the CRB can be obtained by block-wise inversion:

(A7) $$CRB_{\theta }=\frac{1}{N}{\left[M_{\theta }^{H}\left(I-M_{s}{\left(M_{s}^{H}M_{s}\right)}^{-1}M_{s}^{H}\right)M_{\theta }\right]}^{-1}.$$
